# Supplementary material for: An HIV Feedback Resistor: Auto-Regulatory Circuit Deactivator and Noise Buffer
Source: PLoS Biol. 2006 Dec 26;5(1):e9. doi: 10.1371/journal.pbio.0050009 (PMC1717016; doi:10.1371/journal.pbio.0050009)
Supplement: Supplementary file 1 [file pbio.0050009.sg001.pdf]

Fig. S1 Weinberger & Shenk

Equilibria & Stability Analysis for the *Feedback–resistor* model & difference between the *Feedback–resistor* and equilibrium in standard trancriptional rates

---

Here we analyze the Equations 1 and 2 from the the text:

Eq 1 : $\frac{d}{dt}\text{Tat}_D = -k_{p300}\,\text{Tat}_D + (k_{TR} + k_{SIRT1})\,\text{Tat}_A - \delta\,\text{Tat}_D$

Eq. 2 : $\frac{d}{dt}\text{Tat}_A = k_{p300}\,\text{Tat}_D - k_{SIRT1}\,\text{Tat}_A$

which are equivalent to the equations used to generate Fig. 1 *d*.

At steady state solution can be found by setting $\frac{d}{dt}\text{Tat}_D = 0$ then :

$$(k_{TR} + k_{SIRT1})\,\overline{\text{Tat}_A} - (\delta + k_{p300})\,\overline{\text{Tat}_D} = 0$$

and $\frac{d}{dt}\text{Tat}_A = 0$ then :

$$k_{p300}\,\overline{\text{Tat}_D} - k_{SIRT1}\,\overline{\text{Tat}_A} = 0$$

$\therefore\ \overline{\text{Tat}_A} = \overline{\text{Tat}_D} = 0$ is *a* solution (*i.e.* existence is satisfied).
Next we show that this solution is unique.

Stability Analysis and derivation of stability criterion ST1 :

The Jacobian for Eqs. 1 & 2 is:

Printed by Mathematica for Students

$$eq1 = -k_{p300}\ Tat_D + (k_{TR} + k_{SIRT1})\ Tat_A - \delta\ Tat_D;$$
$$eq2 = k_{p300}\ Tat_D - k_{SIRT1}\ Tat_A;$$

$$jacobian = \begin{pmatrix} \partial_{Tat_D} eq1 & \partial_{Tat_A} eq1 \\ \partial_{Tat_D} eq2 & \partial_{Tat_A} eq2 \end{pmatrix};$$

jacobian // MatrixForm

$$\begin{pmatrix} -\delta - k_{p300} & k_{SIRT1} + k_{TR} \\ k_{p300} & -k_{SIRT1} \end{pmatrix}$$

and the determinant is :

jacobian // Det

$$\delta\, k_{SIRT1} - k_{p300}\, k_{TR}$$

Thus, as long as the determinant is $\neq$ 0 or equivalently :

$$\delta\, k_{SIRT1} \neq k_{p300}\, k_{TR}$$

we know that the solution is unique. Below we show that this is indeed the case.

Stability theory states that *a* system of equations is stable if all Eigenvalues of the Jacobian are real and negative equivalentally when the trace is negative and the determinant is positive. The trace of the above matrix is clearly negative and the determinant is positive when :

$$(\delta + k_{p300})\, k_{SIRT1} > (k_{SIRT1} + k_{TR})\, k_{p300}$$

which simplifies to :

$$\delta \times k_{SIRT1} > k_{TR} \times k_{p300}$$

which is the stability criterion ST1.

Below we show explicitly that the eigenvalues are less than zero. the eigenvalues of the jacobian are :

Eigenvalues[jacobian] // Simplify

$$\left\{\frac{1}{2}\left(-\delta - k_{p300} - k_{SIRT1} - \sqrt{-4\,\delta\, k_{SIRT1} + (\delta + k_{p300} + k_{SIRT1})^2 + 4\, k_{p300}\, k_{TR}}\right),\right.$$
$$\left.\frac{1}{2}\left(-\delta - k_{p300} - k_{SIRT1} + \sqrt{-4\,\delta\, k_{SIRT1} + (\delta + k_{p300} + k_{SIRT1})^2 + 4\, k_{p300}\, k_{TR}}\right)\right\}$$

Weinberger & Shenk, Fig. S1

For the all eigenvalues to be negative, the largest eigenvalue needs to be < 0. Thus :

$$\left(-\delta - k_{\mathrm{p300}} - k_{\mathrm{SIRT1}} + \sqrt{-4\,\delta\,k_{\mathrm{SIRT1}} + (\delta + k_{\mathrm{p300}} + k_{\mathrm{SIRT1}})^2 + 4\,k_{\mathrm{p300}}\,k_{\mathrm{TR}}}\right) < 0 \text{ // Simplify}$$

$$\sqrt{-4\,\delta\,k_{\mathrm{SIRT1}} + (\delta + k_{\mathrm{p300}} + k_{\mathrm{SIRT1}})^2 + 4\,k_{\mathrm{p300}}\,k_{\mathrm{TR}}} < \delta + k_{\mathrm{p300}} + k_{\mathrm{SIRT1}}$$

Squaring both sides yields :

$$\left(\sqrt{-4\,\delta\,k_{\mathrm{SIRT1}} + (\delta + k_{\mathrm{p300}} + k_{\mathrm{SIRT1}})^2 + 4\,k_{\mathrm{p300}}\,k_{\mathrm{TR}}}\right)^2 < (\delta + k_{\mathrm{p300}} + k_{\mathrm{SIRT1}})^2 \text{ // Simplify}$$

$$4\,k_{\mathrm{p300}}\,k_{\mathrm{TR}} < 4\,\delta\,k_{\mathrm{SIRT1}}$$

Which cancels to the FINAL answer (which is the stability criterion ST1) :

$$k_{\mathrm{p300}}\,k_{\mathrm{TR}} < \delta\,k_{\mathrm{SIRT1}}$$

Obviously the uniqueness criterion :

$$\delta\,k_{\mathrm{SIRT1}} \neq k_{\mathrm{p300}}\,k_{\mathrm{TR}}$$

is also satisfied in this case.

Furthermore,
since neither Tat_A nor Tat_D appear in the Jacobian (or its determinant), as long as $k_{\mathrm{p300}}\,k_{\mathrm{TR}} < \delta\,k_{\mathrm{SIRT1}}$ is satisfied all steady states are stable.

Finally,
since this is *a* linear system the Jacobian is equivalent to the characteristic matrix of the system (*i.e.* for $\dot{x} = \mathrm{Ax}$, the Jacobian is equal to the matrix A).
For *a* linear system, when the determinant of the matrix A is non − zero (*i.e.* $\delta\,k_{\mathrm{SIRT1}} \neq k_{\mathrm{p300}}\,k_{\mathrm{TR}}$), any solution that exist is unique.

We have thus shown that if $k_{\mathrm{p300}}\,k_{\mathrm{TR}} < \delta\,k_{\mathrm{SIRT1}}$ then $\overline{\mathrm{Tat}_A} = \overline{\mathrm{Tat}_D} = 0$ is the only solution and is globally asymptotically stable (existence and uniqueness).

Incidentally, inserting a GFP "reporter" equation into the system:

$$\text{Eq 1}: \frac{d}{dt}\text{Tat}_D = -k_{p300}\,\text{Tat}_D + (k_{TR} + k_{SIRT1})\,\text{Tat}_A - \delta\,\text{Tat}_D$$

$$\text{Eq. 2}: \frac{d}{dt}\text{Tat}_A = k_{p300}\,\text{Tat}_D - k_{SIRT1}\,\text{Tat}_A$$

$$\text{Eq. 3}: \frac{d}{dt}\text{GFP} = \text{IRES}\,k_{TR}\,\text{Tat}_A - \gamma\,\text{GFP}$$

does not change the zero solution of the system.

```
eq1 = -k_p300 Tat_D + (k_TR + k_SIRT1) Tat_A - δ Tat_D == 0;
eq2 = k_p300 Tat_D - k_SIRT1 Tat_A == 0;
eq3 = IRES k_TR Tat_A - γ GFP == 0;

Solve[{eq1, eq2, eq3}, {Tat_D, Tat_A, GFP}]

{{GFP → 0, Tat_D → 0, Tat_A → 0}}
```

This can be understood since if eq3 = 0 then :

$$\text{IRES}\,k_{TR}\,\overline{\text{Tat}_A} = \gamma\,\overline{\text{GFP}}$$

Since $\overline{\text{Tat}_A} = 0$

$\therefore\ \overline{\text{GFP}} = 0$

Furthermore,
Eq. 3 and the GFP variable do not change the jacobian for Eqs. 1 and 2.
Thus, zero is still the unique solution
of hte system and is globally asymptotically stable.

<u>Difference between a *feedback-resistor* and standard equilibrium rates of transcription</u>

Here we assume that "equilibrium effects" on transcription rates refers to the parameters of mRNA synthesis (i.e. transcriptional initiation, elongation, and termination) and decay. But these processes can be extended to include mRNA splicing, nuclear export, translation and protein decay.

It is a well-documented (and mathematically provable) result that, in general, imbedding intermediate non-decaying reservoirs in an existing system of differential equations, generates a gamma-distributed delay in the system of equations. This is typically referred to as the "box-car" method of introducing a delay, in analogy to box-cars on a train. The delay arrives because it takes time for a response or perturbation in a single compartment or box-car to traverse completely through the entire system of equations or box-cars. The delay is gamma-distributed b/c its effect trickles through the system and reaches any given compartment over time. This can be easily understood since the flow traversing the system of equations must now traverse these extra, added equations as well, and this takes some amount of time for each compartment. In relation to the pinball metaphor, each bump of the "bumpers" takes time, thereby slowing the ball's overall progression down the table. In the extremes, the delay has non-gamma distributions. For example, as the number of added reservoirs approaches infinity, the delay approaches the form of a delta function and the typical "fixed-time" delay can be substituted. That is, the effect of the delay no longer trickles through the system, it's effect arrives at the last compartment all at once. Alternatively, if only a single intermediate compartment is added, the delay is exponentially distributed.

In general, including the transcriptional/translational processes above (without mRNA/protein decay) can only produce a gamma-distributed delay in a gene circuit or a positive-feedback loop (or an exponential or fixed delay). Even a fixed delay (i.e. infinite intermediate compartments) itself cannot stabilize the off-state of a linear, single component positive-feedback loop (this is mathematically provable and is presented in J.D. Murray, <u>Mathematical Biology</u>, Springer, p. 13-21). In relation to the pinball metaphor, if the pinballs are infinitely long-lived then transcriptional processes would increase the length of the table but all pinballs would still eventually drop through the bottom and initiate the positive feedback reaction. Thus, merely including extra transcription steps <u>alone</u> cannot generate a resistor.

But, it is important to note, if decay of the intermediate compartments is considered, in theory, the delay generated by transcriptional processes could indeed comprise a "weak" resistor. Specifically, when mRNA decay is considered and if the mRNA's are very short-lived <u>and</u> the positive-feedback "gain" is small, then transcription/translational processes could indeed constitute an effective weak feedback-resistor. In viral systems, transactivator proteins are exceptionally long-lived, e.g. HIV Tat $t_{1/2}$>8hrs. Thus, "equilibrium effects on transcription rates" cannot themselves comprise a sufficiently strong feedback-resistor in these systems. In other systems with short-lived transactivator species, standard transcriptional processes may indeed comprise a feedback-resistor.
