# Supplementary material for: An HIV Feedback Resistor: Auto-Regulatory Circuit Deactivator and Noise Buffer
Source: PLoS Biol. 2006 Dec 26;5(1):e9. doi: 10.1371/journal.pbio.0050009 (PMC1717016; doi:10.1371/journal.pbio.0050009)
Supplement: Supplementary file 2 [file pbio.0050009.sg002.pdf]

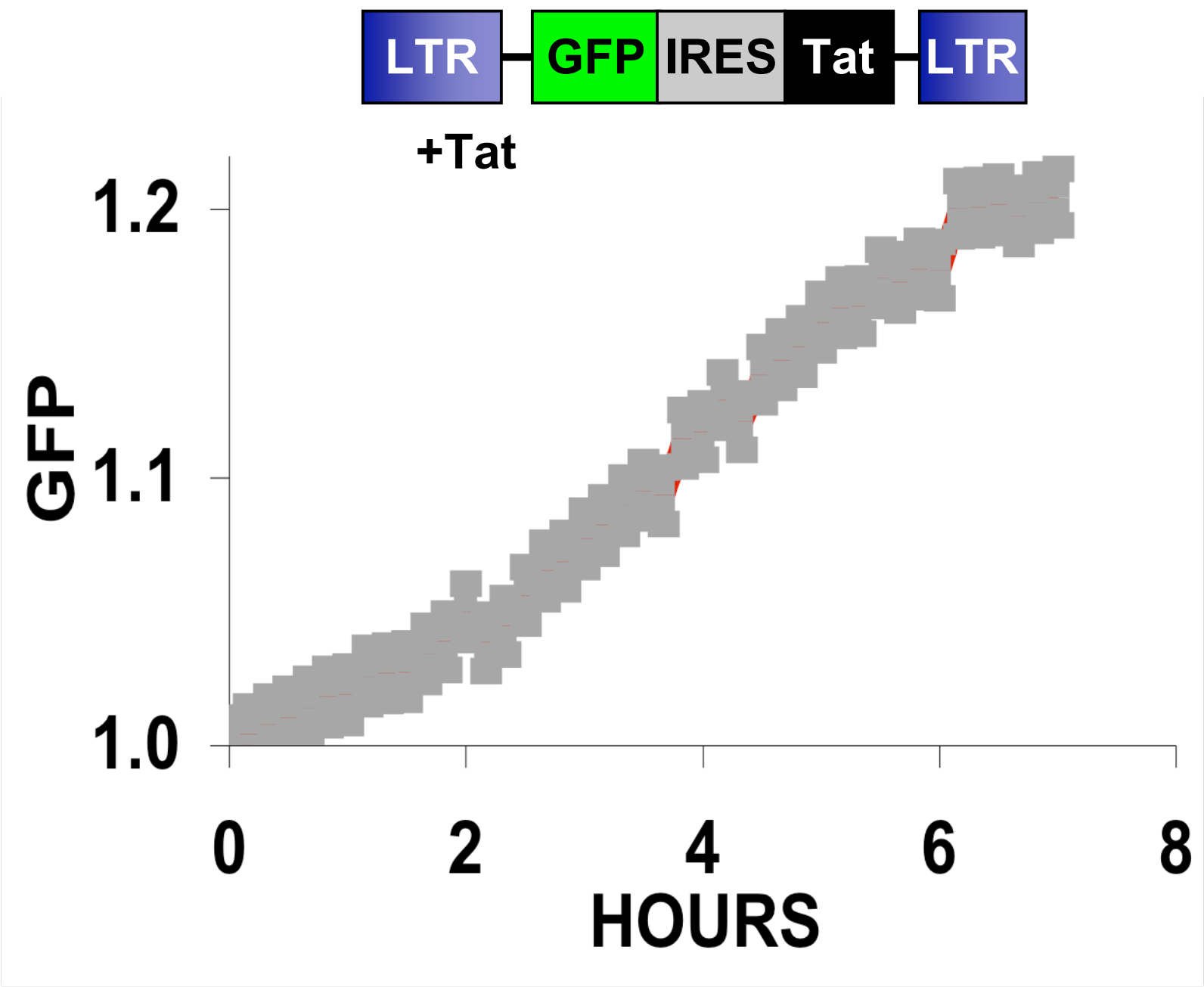
LTR
GFP
IRES
Tat
LTR
+Tat
GFP
1.2
1.1
1.0
0
2
4
6
8
HOURS

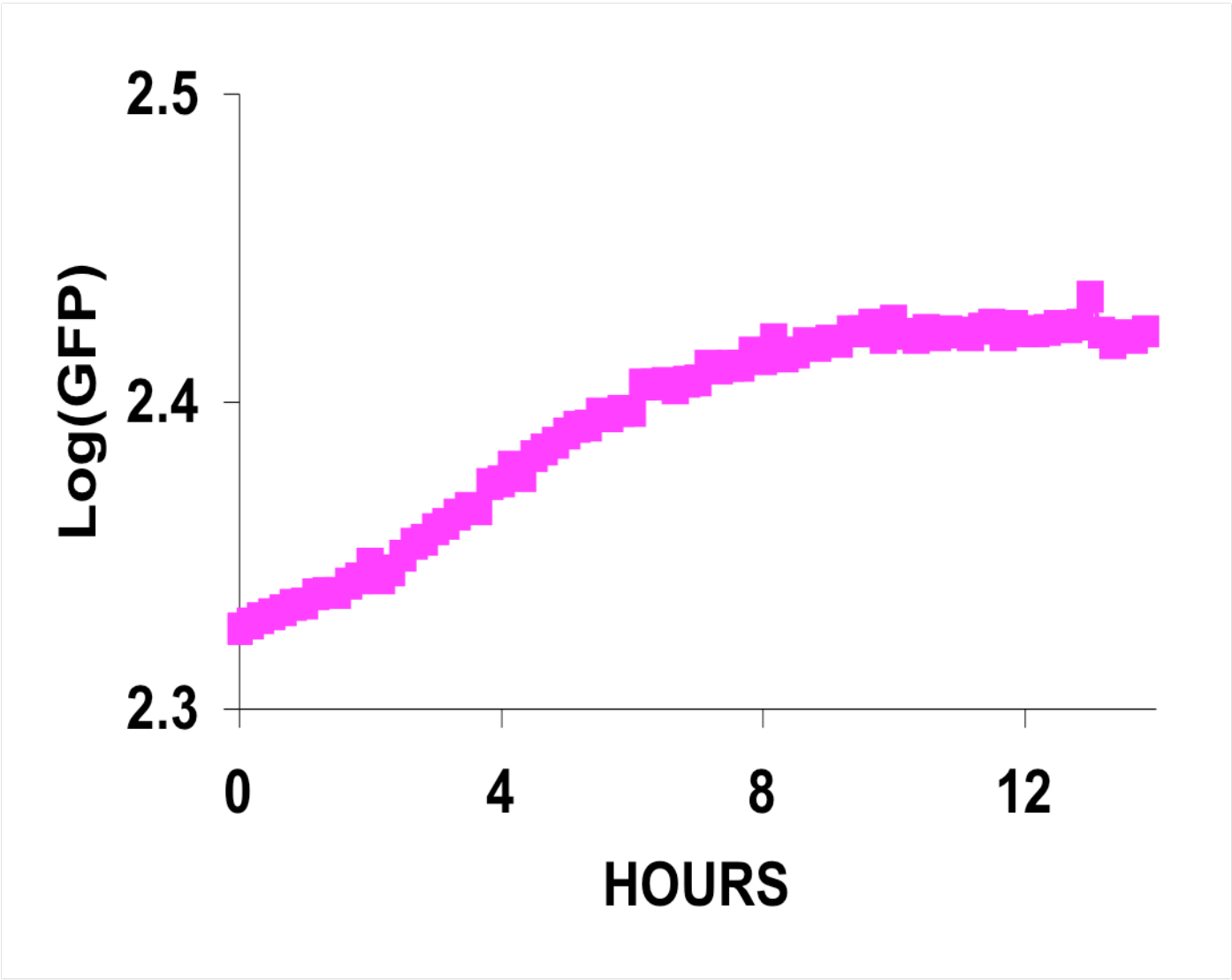
Log(GFP)
2.5
2.4
2.3
0
4
8
12
HOURS
