# Supplementary material for: An HIV Feedback Resistor: Auto-Regulatory Circuit Deactivator and Noise Buffer
Source: PLoS Biol. 2006 Dec 26;5(1):e9. doi: 10.1371/journal.pbio.0050009 (PMC1717016; doi:10.1371/journal.pbio.0050009)
Supplement: Supplementary file 3 [file pbio.0050009.sg003.pdf]

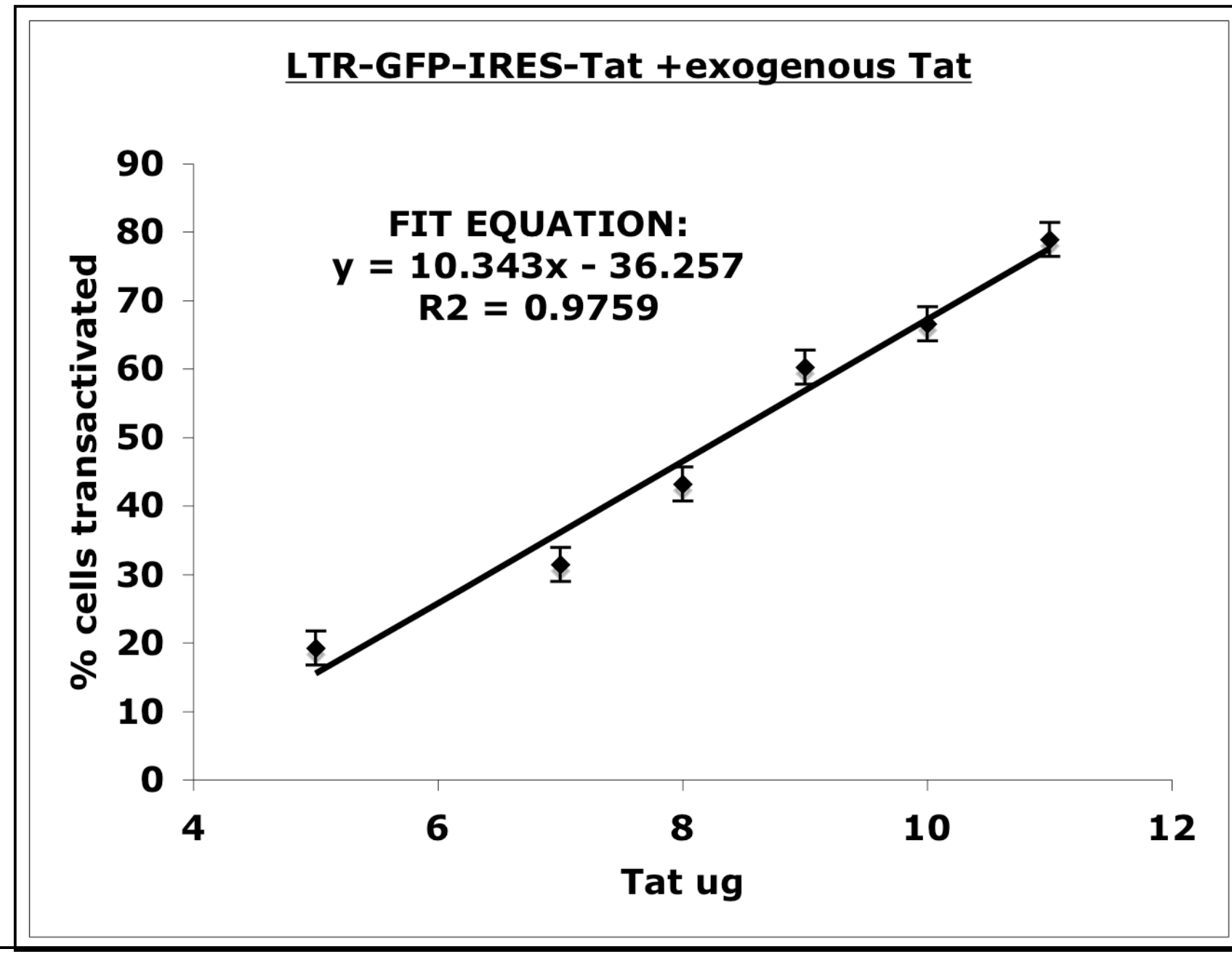
LTR-GFP-IRES-Tat +exogenous Tat
FIT EQUATION:
y = 10.343x - 36.257
R2 = 0.9759
% cells transactivated
90
80
70
60
50
40
30
20
10
0
4
6
8
10
12
Tat ug

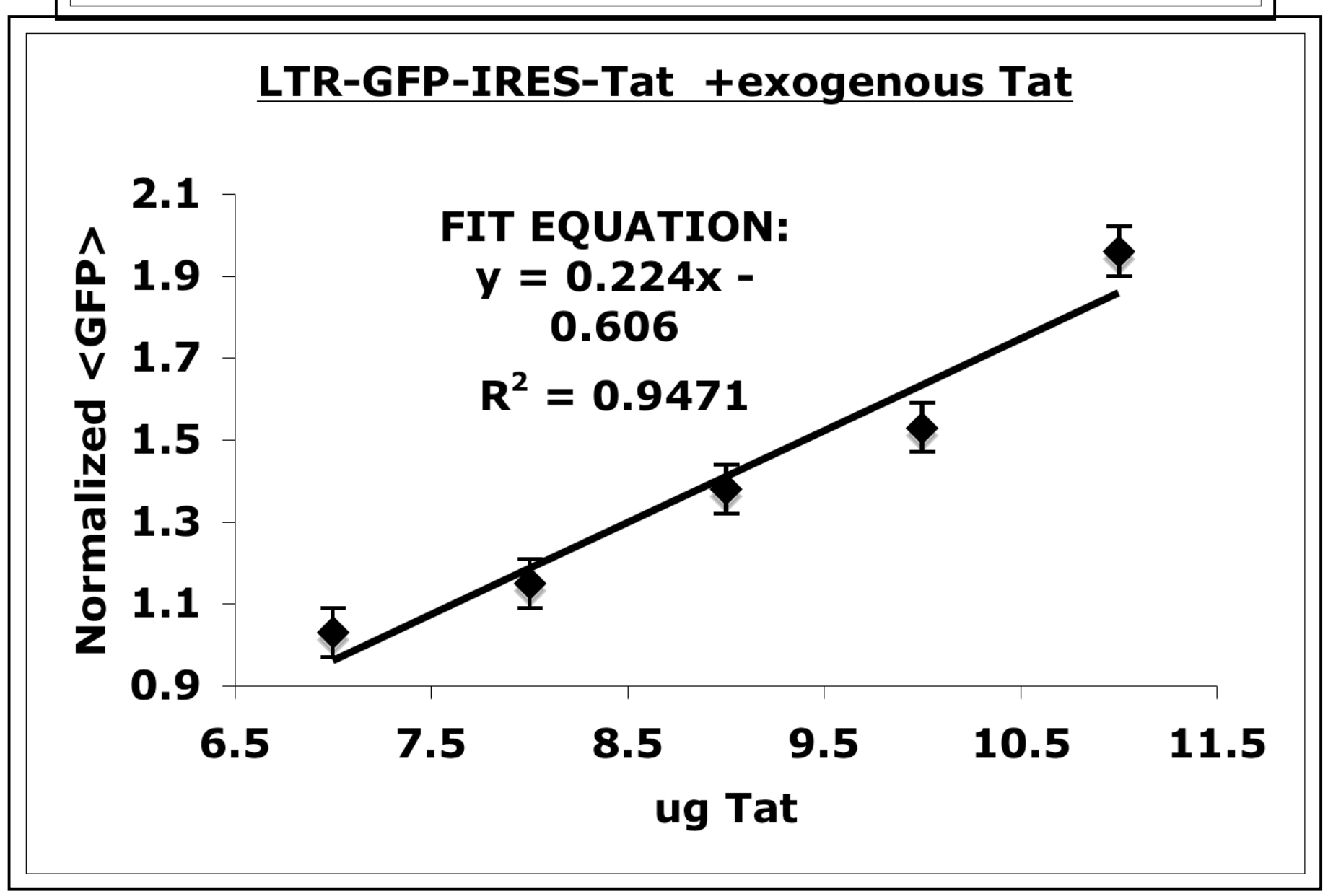
LTR-GFP-IRES-Tat +exogenous Tat
FIT EQUATION:
y = 0.224x -
0.606
R2 = 0.9471
Normalized <GFP>
2.1
1.9
1.7
1.5
1.3
1.1
0.9
6.5
7.5
8.5
9.5
10.5
11.5
ug Tat
