# Supplementary material for: An HIV Feedback Resistor: Auto-Regulatory Circuit Deactivator and Noise Buffer
Source: PLoS Biol. 2006 Dec 26;5(1):e9. doi: 10.1371/journal.pbio.0050009 (PMC1717016; doi:10.1371/journal.pbio.0050009)
Supplement: Supplementary file 4 [file pbio.0050009.sg004.pdf]

Analytical Solutions to Eqs.1 & 2

$$\Big\{\Big\{\mathrm{Tat}_D(t) \to$$

$$\Big(e^{-\frac{1}{2}t\left(\delta - k_{\mathrm{p300}} + k_{\mathrm{SIRT1}} + \sqrt{k_{\mathrm{p300}}^2 + (-2\delta + 6k_{\mathrm{SIRT1}} + 4k_{\mathrm{TR}})k_{\mathrm{p300}} + (\delta - k_{\mathrm{SIRT1}})^2}\right)}\Big(-e^{t\sqrt{k_{\mathrm{p300}}^2 + (-2\delta + 6k_{\mathrm{SIRT1}} + 4k_{\mathrm{TR}})k_{\mathrm{p300}} + (\delta - k_{\mathrm{SIRT1}})^2}}$$

$$\delta\, c_1 + \delta\, c_1 + \left(-1 + e^{t\sqrt{k_{\mathrm{p300}}^2 + (-2\delta + 6k_{\mathrm{SIRT1}} + 4k_{\mathrm{TR}})k_{\mathrm{p300}} + (\delta - k_{\mathrm{SIRT1}})^2}}\right) k_{\mathrm{p300}}\, c_1 +$$

$$e^{t\sqrt{k_{\mathrm{p300}}^2 + (-2\delta + 6k_{\mathrm{SIRT1}} + 4k_{\mathrm{TR}})k_{\mathrm{p300}} + (\delta - k_{\mathrm{SIRT1}})^2}} \sqrt{(k_{\mathrm{p300}}^2 + (-2\delta + 6k_{\mathrm{SIRT1}} + 4k_{\mathrm{TR}})k_{\mathrm{p300}} +}$$

$$(\delta - k_{\mathrm{SIRT1}})^2)\, c_1 + \sqrt{(k_{\mathrm{p300}}^2 + (-2\delta + 6k_{\mathrm{SIRT1}} + 4k_{\mathrm{TR}})k_{\mathrm{p300}} + (\delta - k_{\mathrm{SIRT1}})^2)}\, c_1 +$$

$$\left(-1 + e^{t\sqrt{k_{\mathrm{p300}}^2 + (-2\delta + 6k_{\mathrm{SIRT1}} + 4k_{\mathrm{TR}})k_{\mathrm{p300}} + (\delta - k_{\mathrm{SIRT1}})^2}}\right)(c_1 + 2c_2)\, k_{\mathrm{SIRT1}} +$$

$$2\, e^{t\sqrt{k_{\mathrm{p300}}^2 + (-2\delta + 6k_{\mathrm{SIRT1}} + 4k_{\mathrm{TR}})k_{\mathrm{p300}} + (\delta - k_{\mathrm{SIRT1}})^2}}\, c_2\, k_{\mathrm{TR}} - 2\, c_2\, k_{\mathrm{TR}}\Big)\Big) \Big/$$

$$(2\sqrt{(k_{\mathrm{p300}}^2 + (-2\delta + 6k_{\mathrm{SIRT1}} + 4k_{\mathrm{TR}})k_{\mathrm{p300}} + (\delta - k_{\mathrm{SIRT1}})^2)}),$$

$$\mathrm{Tat}_A(t) \to \Big(e^{-\frac{1}{2}t\left(\delta - k_{\mathrm{p300}} + k_{\mathrm{SIRT1}} + \sqrt{k_{\mathrm{p300}}^2 + (-2\delta + 6k_{\mathrm{SIRT1}} + 4k_{\mathrm{TR}})k_{\mathrm{p300}} + (\delta - k_{\mathrm{SIRT1}})^2}\right)}$$

$$\Big(\Big(-1 + e^{t\sqrt{k_{\mathrm{p300}}^2 + (-2\delta + 6k_{\mathrm{SIRT1}} + 4k_{\mathrm{TR}})k_{\mathrm{p300}} + (\delta - k_{\mathrm{SIRT1}})^2}}\Big)(2c_1 - c_2)\, k_{\mathrm{p300}} +$$

$$c_2\Big(\sqrt{(k_{\mathrm{p300}}^2 + (-2\delta + 6k_{\mathrm{SIRT1}} + 4k_{\mathrm{TR}})k_{\mathrm{p300}} + (\delta - k_{\mathrm{SIRT1}})^2)}$$

$$\left(1 + e^{t\sqrt{k_{\mathrm{p300}}^2 + (-2\delta + 6k_{\mathrm{SIRT1}} + 4k_{\mathrm{TR}})k_{\mathrm{p300}} + (\delta - k_{\mathrm{SIRT1}})^2}}\right) +$$

$$\left(-1 + e^{t\sqrt{k_{\mathrm{p300}}^2 + (-2\delta + 6k_{\mathrm{SIRT1}} + 4k_{\mathrm{TR}})k_{\mathrm{p300}} + (\delta - k_{\mathrm{SIRT1}})^2}}\right)\delta -$$

$$\left(-1 + e^{t\sqrt{k_{\mathrm{p300}}^2 + (-2\delta + 6k_{\mathrm{SIRT1}} + 4k_{\mathrm{TR}})k_{\mathrm{p300}} + (\delta - k_{\mathrm{SIRT1}})^2}}\right)k_{\mathrm{SIRT1}}\Big)\Big)\Big) \Big/$$

$$(2\sqrt{(k_{\mathrm{p300}}^2 + (-2\delta + 6k_{\mathrm{SIRT1}} + 4k_{\mathrm{TR}})k_{\mathrm{p300}} + (\delta - k_{\mathrm{SIRT1}})^2)})\Big\}\Big\}$$

When an equation for GFP :

$$\frac{d}{dt}\,GFP = IRES \times k_{TR} \times Tat_A - \gamma\,GFP$$

is included in the system of equations the solutions for $Tat_D(t)$ and $Tat_A(t)$ do not change and the solution for $GFP(t)$ is :

$$\mathrm{GFP}(t) \to \Bigg(e^{-\frac{1}{2}t\left(\delta-k_{\mathrm{p300}}+k_{\mathrm{SIRT1}}+\sqrt{k_{\mathrm{p300}}^2+(-2\delta+6k_{\mathrm{SIRT1}}+4k_{\mathrm{TR}})k_{\mathrm{p300}}+(\delta-k_{\mathrm{SIRT1}})^2}\right)}$$

$$\Bigg(\Bigg(2e^{\frac{1}{2}t\left(-2\gamma+\delta-k_{\mathrm{p300}}+k_{\mathrm{SIRT1}}+\sqrt{k_{\mathrm{p300}}^2+(-2\delta+6k_{\mathrm{SIRT1}}+4k_{\mathrm{TR}})k_{\mathrm{p300}}+(\delta-k_{\mathrm{SIRT1}})^2}\right)}\gamma c_3 -$$

$$4e^{\frac{1}{2}t\left(-2\gamma+\delta-k_{\mathrm{p300}}+k_{\mathrm{SIRT1}}+\sqrt{k_{\mathrm{p300}}^2+(-2\delta+6k_{\mathrm{SIRT1}}+4k_{\mathrm{TR}})k_{\mathrm{p300}}+(\delta-k_{\mathrm{SIRT1}})^2}\right)}k_{\mathrm{SIRT1}}\,c_3 +$$

$$\Bigg(-\Bigg(1+e^{t\sqrt{k_{\mathrm{p300}}^2+(-2\delta+6k_{\mathrm{SIRT1}}+4k_{\mathrm{TR}})k_{\mathrm{p300}}+(\delta-k_{\mathrm{SIRT1}})^2}} -$$

$$2e^{\frac{1}{2}t\left(-2\gamma+\delta-k_{\mathrm{p300}}+k_{\mathrm{SIRT1}}+\sqrt{k_{\mathrm{p300}}^2+(-2\delta+6k_{\mathrm{SIRT1}}+4k_{\mathrm{TR}})k_{\mathrm{p300}}+(\delta-k_{\mathrm{SIRT1}})^2}\right)}\Bigg)\mathrm{IRES}$$

$$(c_1-c_2)-2e^{\frac{1}{2}t\left(-2\gamma+\delta-k_{\mathrm{p300}}+k_{\mathrm{SIRT1}}+\sqrt{k_{\mathrm{p300}}^2+(-2\delta+6k_{\mathrm{SIRT1}}+4k_{\mathrm{TR}})k_{\mathrm{p300}}+(\delta-k_{\mathrm{SIRT1}})^2}\right)}$$

$$c_3\Bigg)k_{\mathrm{TR}}\Bigg)k_{\mathrm{p300}}^3 +$$

$$\Bigg(-24e^{\frac{1}{2}t\left(-2\gamma+\delta-k_{\mathrm{p300}}+k_{\mathrm{SIRT1}}+\sqrt{k_{\mathrm{p300}}^2+(-2\delta+6k_{\mathrm{SIRT1}}+4k_{\mathrm{TR}})k_{\mathrm{p300}}+(\delta-k_{\mathrm{SIRT1}})^2}\right)}c_3\,k_{\mathrm{SIRT1}}^2 +$$

$$2\Bigg(5e^{\frac{1}{2}t\left(-2\gamma+\delta-k_{\mathrm{p300}}+k_{\mathrm{SIRT1}}+\sqrt{k_{\mathrm{p300}}^2+(-2\delta+6k_{\mathrm{SIRT1}}+4k_{\mathrm{TR}})k_{\mathrm{p300}}+(\delta-k_{\mathrm{SIRT1}})^2}\right)}(\gamma+\delta)c_3 +$$

$$\Bigg(-3\Bigg(1+e^{t\sqrt{k_{\mathrm{p300}}^2+(-2\delta+6k_{\mathrm{SIRT1}}+4k_{\mathrm{TR}})k_{\mathrm{p300}}+(\delta-k_{\mathrm{SIRT1}})^2}} -$$

$$2e^{\frac{1}{2}t\left(-2\gamma+\delta-k_{\mathrm{p300}}+k_{\mathrm{SIRT1}}+\sqrt{k_{\mathrm{p300}}^2+(-2\delta+6k_{\mathrm{SIRT1}}+4k_{\mathrm{TR}})k_{\mathrm{p300}}+(\delta-k_{\mathrm{SIRT1}})^2}\right)}\Bigg)$$

$$\mathrm{IRES}(c_1-c_2)-14$$

$$e^{\frac{1}{2}t\left(-2\gamma+\delta-k_{\mathrm{p300}}+k_{\mathrm{SIRT1}}+\sqrt{k_{\mathrm{p300}}^2+(-2\delta+6k_{\mathrm{SIRT1}}+4k_{\mathrm{TR}})k_{\mathrm{p300}}+(\delta-k_{\mathrm{SIRT1}})^2}\right)}c_3\Bigg)k_{\mathrm{TR}}\Bigg)$$

$$k_{\mathrm{SIRT1}}-4\Bigg(\Bigg(\Bigg(1+e^{t\sqrt{k_{\mathrm{p300}}^2+(-2\delta+6k_{\mathrm{SIRT1}}+4k_{\mathrm{TR}})k_{\mathrm{p300}}+(\delta-k_{\mathrm{SIRT1}})^2}}-2$$

$$e^{\frac{1}{2}t\left(-2\gamma+\delta-k_{\mathrm{p300}}+k_{\mathrm{SIRT1}}+\sqrt{k_{\mathrm{p300}}^2+(-2\delta+6k_{\mathrm{SIRT1}}+4k_{\mathrm{TR}})k_{\mathrm{p300}}+(\delta-k_{\mathrm{SIRT1}})^2}\right)}\Bigg)\mathrm{IRES}$$

$$(c_1-c_2)+2e^{\frac{1}{2}t\left(-2\gamma+\delta-k_{\mathrm{p300}}+k_{\mathrm{SIRT1}}+\sqrt{k_{\mathrm{p300}}^2+(-2\delta+6k_{\mathrm{SIRT1}}+4k_{\mathrm{TR}})k_{\mathrm{p300}}+(\delta-k_{\mathrm{SIRT1}})^2}\right)}c_3\Bigg)$$

$$k_{\mathrm{TR}}^2+2e^{\frac{1}{2}t\left(-2\gamma+\delta-k_{\mathrm{p300}}+k_{\mathrm{SIRT1}}+\sqrt{k_{\mathrm{p300}}^2+(-2\delta+6k_{\mathrm{SIRT1}}+4k_{\mathrm{TR}})k_{\mathrm{p300}}+(\delta-k_{\mathrm{SIRT1}})^2}\right)}\gamma(\gamma-3\delta)c_3 +$$

$$k_{\mathrm{TR}}\Bigg(4e^{\frac{1}{2}t\left(-2\gamma+\delta-k_{\mathrm{p300}}+k_{\mathrm{SIRT1}}+\sqrt{k_{\mathrm{p300}}^2+(-2\delta+6k_{\mathrm{SIRT1}}+4k_{\mathrm{TR}})k_{\mathrm{p300}}+(\delta-k_{\mathrm{SIRT1}})^2}\right)}(2\gamma+\delta)c_3 +$$

$$\mathrm{IRES}\Bigg(\Bigg(\Big(1+e^{t\sqrt{k_{\mathrm{p300}}^2+(-2\delta+6k_{\mathrm{SIRT1}}+4k_{\mathrm{TR}})k_{\mathrm{p300}}+(\delta-k_{\mathrm{SIRT1}})^2}}-$$

$$2e^{\frac{1}{2}t\left(-2\gamma+\delta-k_{\mathrm{p300}}+k_{\mathrm{SIRT1}}+\sqrt{k_{\mathrm{p300}}^2+(-2\delta+6k_{\mathrm{SIRT1}}+4k_{\mathrm{TR}})k_{\mathrm{p300}}+(\delta-k_{\mathrm{SIRT1}})^2}\right)}\Big)\delta$$

$$(2c_1-3c_2)+\Big(1+e^{t\sqrt{k_{\mathrm{p300}}^2+(-2\delta+6k_{\mathrm{SIRT1}}+4k_{\mathrm{TR}})k_{\mathrm{p300}}+(\delta-k_{\mathrm{SIRT1}})^2}}-$$

$$2e^{\frac{1}{2}t\left(-2\gamma+\delta-k_{\mathrm{p300}}+k_{\mathrm{SIRT1}}+\sqrt{k_{\mathrm{p300}}^2+(-2\delta+6k_{\mathrm{SIRT1}}+4k_{\mathrm{TR}})k_{\mathrm{p300}}+(\delta-k_{\mathrm{SIRT1}})^2}\right)}\Big)\gamma$$

$$c_2+\Big(-1+e^{t\sqrt{k_{\mathrm{p300}}^2+(-2\delta+6k_{\mathrm{SIRT1}}+4k_{\mathrm{TR}})k_{\mathrm{p300}}+(\delta-k_{\mathrm{SIRT1}})^2}}\Big)(c_1-c_2)$$

$$\sqrt{(k_{\mathrm{p300}}^2+(-2\delta+6k_{\mathrm{SIRT1}}+4k_{\mathrm{TR}})k_{\mathrm{p300}}+(\delta-k_{\mathrm{SIRT1}})^2)}\Bigg)\Bigg)k_{\mathrm{p300}}^2-$$

$$\Bigg(4e^{\frac{1}{2}t\left(-2\gamma+\delta-k_{\mathrm{p300}}+k_{\mathrm{SIRT1}}+\sqrt{k_{\mathrm{p300}}^2+(-2\delta+6k_{\mathrm{SIRT1}}+4k_{\mathrm{TR}})k_{\mathrm{p300}}+(\delta-k_{\mathrm{SIRT1}})^2}\right)}c_3k_{\mathrm{SIRT1}}^3+$$

$$\Bigg(10e^{\frac{1}{2}t\left(-2\gamma+\delta-k_{\mathrm{p300}}+k_{\mathrm{SIRT1}}+\sqrt{k_{\mathrm{p300}}^2+(-2\delta+6k_{\mathrm{SIRT1}}+4k_{\mathrm{TR}})k_{\mathrm{p300}}+(\delta-k_{\mathrm{SIRT1}})^2}\right)}(\gamma-2\delta)c_3+$$

$$\Bigg(\Big(1+e^{t\sqrt{k_{\mathrm{p300}}^2+(-2\delta+6k_{\mathrm{SIRT1}}+4k_{\mathrm{TR}})k_{\mathrm{p300}}+(\delta-k_{\mathrm{SIRT1}})^2}}-$$

$$2e^{\frac{1}{2}t\left(-2\gamma+\delta-k_{\mathrm{p300}}+k_{\mathrm{SIRT1}}+\sqrt{k_{\mathrm{p300}}^2+(-2\delta+6k_{\mathrm{SIRT1}}+4k_{\mathrm{TR}})k_{\mathrm{p300}}+(\delta-k_{\mathrm{SIRT1}})^2}\right)}\Big)\mathrm{IRES}$$

$$(c_1-c_2)+2e^{\frac{1}{2}t\left(-2\gamma+\delta-k_{\mathrm{p300}}+k_{\mathrm{SIRT1}}+\sqrt{k_{\mathrm{p300}}^2+(-2\delta+6k_{\mathrm{SIRT1}}+4k_{\mathrm{TR}})k_{\mathrm{p300}}+(\delta-k_{\mathrm{SIRT1}})^2}\right)}$$

$$c_3\Bigg)k_{\mathrm{TR}}\Bigg)k_{\mathrm{SIRT1}}^2+$$

$$\Bigg(k_{\mathrm{TR}}\Bigg(4e^{\frac{1}{2}t\left(-2\gamma+\delta-k_{\mathrm{p300}}+k_{\mathrm{SIRT1}}+\sqrt{k_{\mathrm{p300}}^2+(-2\delta+6k_{\mathrm{SIRT1}}+4k_{\mathrm{TR}})k_{\mathrm{p300}}+(\delta-k_{\mathrm{SIRT1}})^2}\right)}$$

$$(2\gamma-3\delta)c_3+\mathrm{IRES}\Bigg(-2\Big(1+e^{t\sqrt{k_{\mathrm{p300}}^2+(-2\delta+6k_{\mathrm{SIRT1}}+4k_{\mathrm{TR}})k_{\mathrm{p300}}+(\delta-k_{\mathrm{SIRT1}})^2}}-$$

$$2e^{\frac{1}{2}t\left(-2\gamma+\delta-k_{\mathrm{p300}}+k_{\mathrm{SIRT1}}+\sqrt{k_{\mathrm{p300}}^2+(-2\delta+6k_{\mathrm{SIRT1}}+4k_{\mathrm{TR}})k_{\mathrm{p300}}+(\delta-k_{\mathrm{SIRT1}})^2}\right)}\Big)$$

$$\delta(c_1-4c_2)-6\Big(1+e^{t\sqrt{k_{\mathrm{p300}}^2+(-2\delta+6k_{\mathrm{SIRT1}}+4k_{\mathrm{TR}})k_{\mathrm{p300}}+(\delta-k_{\mathrm{SIRT1}})^2}}-$$

$$2e^{\frac{1}{2}t\left(-2\gamma+\delta-k_{\mathrm{p300}}+k_{\mathrm{SIRT1}}+\sqrt{k_{\mathrm{p300}}^2+(-2\delta+6k_{\mathrm{SIRT1}}+4k_{\mathrm{TR}})k_{\mathrm{p300}}+(\delta-k_{\mathrm{SIRT1}})^2}\right)}\Big)$$

$$\gamma c_2+\Big(-1+e^{t\sqrt{k_{\mathrm{p300}}^2+(-2\delta+6k_{\mathrm{SIRT1}}+4k_{\mathrm{TR}})k_{\mathrm{p300}}+(\delta-k_{\mathrm{SIRT1}})^2}}\Big)(c_1+3c_2)$$

$$\sqrt{(k_{\mathrm{p300}}^2+(-2\delta+6k_{\mathrm{SIRT1}}+4k_{\mathrm{TR}})k_{\mathrm{p300}}+(\delta-k_{\mathrm{SIRT1}})^2)}\Bigg)\Bigg)-$$

$$4e^{\frac{1}{2}t\left(-2\gamma+\delta-k_{\mathrm{p300}}+k_{\mathrm{SIRT1}}+\sqrt{k_{\mathrm{p300}}^2+(-2\delta+6k_{\mathrm{SIRT1}}+4k_{\mathrm{TR}})k_{\mathrm{p300}}+(\delta-k_{\mathrm{SIRT1}})^2}\right)}$$

$$(3\gamma^2-3\delta\gamma-2\delta^2)c_3\Bigg)k_{\mathrm{SIRT1}}+$$

$$2e^{\frac{1}{2}t\left(-2\gamma+\delta-k_{\mathrm{p300}}+k_{\mathrm{SIRT1}}+\sqrt{k_{\mathrm{p300}}^2+(-2\delta+6k_{\mathrm{SIRT1}}+4k_{\mathrm{TR}})k_{\mathrm{p300}}+(\delta-k_{\mathrm{SIRT1}})^2}\right)}\gamma(2\gamma-3\delta)\delta c_3+$$

$$2\,\mathrm{IRES}\,c_2k_{\mathrm{TR}}^2\Bigg(\sqrt{(k_{\mathrm{p300}}^2+(-2\delta+6k_{\mathrm{SIRT1}}+4k_{\mathrm{TR}})k_{\mathrm{p300}}+(\delta-k_{\mathrm{SIRT1}})^2)}$$

$$\Big(-1+e^{t\sqrt{k_{\mathrm{p300}}^2+(-2\delta+6k_{\mathrm{SIRT1}}+4k_{\mathrm{TR}})k_{\mathrm{p300}}+(\delta-k_{\mathrm{SIRT1}})^2}}\Big)-$$

$$2\left(1+e^{t\sqrt{k_{p300}^2+(-2\delta+6k_{SIRT1}+4k_{TR})k_{p300}+(\delta-k_{SIRT1})^2}}-2\right.$$
$$\left.e^{\frac{1}{2}t\left(-2\gamma+\delta-k_{p300}+k_{SIRT1}+\sqrt{k_{p300}^2+(-2\delta+6k_{SIRT1}+4k_{TR})k_{p300}+(\delta-k_{SIRT1})^2}\right)}\right)\gamma+$$
$$2\left(1+e^{t\sqrt{k_{p300}^2+(-2\delta+6k_{SIRT1}+4k_{TR})k_{p300}+(\delta-k_{SIRT1})^2}}-2\right.$$
$$\left.\left.e^{\frac{1}{2}t\left(-2\gamma+\delta-k_{p300}+k_{SIRT1}+\sqrt{k_{p300}^2+(-2\delta+6k_{SIRT1}+4k_{TR})k_{p300}+(\delta-k_{SIRT1})^2}\right)}\right)\delta\right)+$$
$$k_{TR}\left(\mathrm{IRES}\left(\left(1+e^{t\sqrt{k_{p300}^2+(-2\delta+6k_{SIRT1}+4k_{TR})k_{p300}+(\delta-k_{SIRT1})^2}}-\right.\right.\right.$$
$$\left.2\,e^{\frac{1}{2}t\left(-2\gamma+\delta-k_{p300}+k_{SIRT1}+\sqrt{k_{p300}^2+(-2\delta+6k_{SIRT1}+4k_{TR})k_{p300}+(\delta-k_{SIRT1})^2}\right)}\right)$$
$$(c_1-3c_2)\,\delta^2+\left(\left(-1+e^{t\sqrt{k_{p300}^2+(-2\delta+6k_{SIRT1}+4k_{TR})k_{p300}+(\delta-k_{SIRT1})^2}}\right)\right.$$
$$\sqrt{(k_{p300}^2+(-2\delta+6k_{SIRT1}+4k_{TR})k_{p300}+(\delta-k_{SIRT1})^2)}$$
$$(c_1-2c_2)+2\left(1+e^{t\sqrt{k_{p300}^2+(-2\delta+6k_{SIRT1}+4k_{TR})k_{p300}+(\delta-k_{SIRT1})^2}}-\right.$$
$$\left.2\,e^{\frac{1}{2}t\left(-2\gamma+\delta-k_{p300}+k_{SIRT1}+\sqrt{k_{p300}^2+(-2\delta+6k_{SIRT1}+4k_{TR})k_{p300}+(\delta-k_{SIRT1})^2}\right)}\right)$$
$$\left.\gamma\,c_2\right)\delta-\left(-1+e^{t\sqrt{k_{p300}^2+(-2\delta+6k_{SIRT1}+4k_{TR})k_{p300}+(\delta-k_{SIRT1})^2}}\right)\gamma$$
$$\left.(2c_1-c_2)\sqrt{(k_{p300}^2+(-2\delta+6k_{SIRT1}+4k_{TR})k_{p300}+(\delta-k_{SIRT1})^2)}\right)-$$
$$2\,e^{\frac{1}{2}t\left(-2\gamma+\delta-k_{p300}+k_{SIRT1}+\sqrt{k_{p300}^2+(-2\delta+6k_{SIRT1}+4k_{TR})k_{p300}+(\delta-k_{SIRT1})^2}\right)}$$
$$\left.\left.(4\gamma^2-4\delta\gamma-\delta^2)\,c_3\right)\right)k_{p300}+(\gamma-\delta)(\delta-k_{SIRT1})$$
$$\left(2\,e^{\frac{1}{2}t\left(-2\gamma+\delta-k_{p300}+k_{SIRT1}+\sqrt{k_{p300}^2+(-2\delta+6k_{SIRT1}+4k_{TR})k_{p300}+(\delta-k_{SIRT1})^2}\right)}c_3\,k_{SIRT1}^2-\right.$$
$$\left(2\,e^{\frac{1}{2}t\left(-2\gamma+\delta-k_{p300}+k_{SIRT1}+\sqrt{k_{p300}^2+(-2\delta+6k_{SIRT1}+4k_{TR})k_{p300}+(\delta-k_{SIRT1})^2}\right)}(\gamma+\delta)\,c_3+\right.$$
$$\left(1+e^{t\sqrt{k_{p300}^2+(-2\delta+6k_{SIRT1}+4k_{TR})k_{p300}+(\delta-k_{SIRT1})^2}}-2\right.$$
$$\left.e^{\frac{1}{2}t\left(-2\gamma+\delta-k_{p300}+k_{SIRT1}+\sqrt{k_{p300}^2+(-2\delta+6k_{SIRT1}+4k_{TR})k_{p300}+(\delta-k_{SIRT1})^2}\right)}\right)\mathrm{IRES}\,c_2$$
$$\left.k_{TR}\right)k_{SIRT1}+2\,e^{\frac{1}{2}t\left(-2\gamma+\delta-k_{p300}+k_{SIRT1}+\sqrt{k_{p300}^2+(-2\delta+6k_{SIRT1}+4k_{TR})k_{p300}+(\delta-k_{SIRT1})^2}\right)}$$
$$\gamma\,\delta\,c_3+\mathrm{IRES}\,c_2\,k_{TR}\left(\sqrt{(k_{p300}^2+(-2\delta+6k_{SIRT1}+4k_{TR})k_{p300}+(\delta-k_{SIRT1})^2)}\right.$$
$$\left(-1+e^{t\sqrt{k_{p300}^2+(-2\delta+6k_{SIRT1}+4k_{TR})k_{p300}+(\delta-k_{SIRT1})^2}}\right)+$$
$$\left(1+e^{t\sqrt{k_{p300}^2+(-2\delta+6k_{SIRT1}+4k_{TR})k_{p300}+(\delta-k_{SIRT1})^2}}-2\right.$$
$$\left.\left.\left.\left.\left.e^{\frac{1}{2}t\left(-2\gamma+\delta-k_{p300}+k_{SIRT1}+\sqrt{k_{p300}^2+(-2\delta+6k_{SIRT1}+4k_{TR})k_{p300}+(\delta-k_{SIRT1})^2}\right)}\right)\delta\right)\right)\right)\right)\Big/$$
$$(2((\gamma-\delta)(\gamma-k_{SIRT1})+k_{p300}(\gamma-2k_{SIRT1}-k_{TR})))$$

$$(k_{p300}^2 + (-2\delta + 6k_{SIRT1} + 4k_{TR}) k_{p300} + (\delta - k_{SIRT1})^2))$$

For simplicity we can substitute numerical values into the above solutions to obtain the following:

Simplify[gfpsolns //. {k_{p300} → .01, k_{SIRT1} → 1, k_{TR} → .1, δ → 2, γ → 0.5, IRES → 10}]

$$\{\{\text{Tat}_D(t) \to e^{-2.00099\,t}\,((0.989141 + 0.0108591\,e^{1.01198\,t})\,c_1 + (-1.08698 + 1.08698\,e^{1.01198\,t})\,c_2),$$
$$\text{Tat}_A(t) \to e^{-2.00099\,t}\,((-0.00988164 + 0.00988164\,e^{1.01198\,t})\,c_1 + (0.0108591 + 0.989141\,e^{1.01198\,t})\,c_2),$$
$$\text{GFP}(t) \to e^{-2.00099\,t}\,((0.00658342 - 0.0202074\,e^{1.01198\,t} + 0.013624\,e^{1.50099\,t})\,c_1 + (-0.0072346 - 2.02274\,e^{1.01198\,t} + 2.02997\,e^{1.50099\,t})\,c_2 + 1.\,e^{1.50099\,t}\,c_3)\}\}$$
