# Supplementary material for: An HIV Feedback Resistor: Auto-Regulatory Circuit Deactivator and Noise Buffer
Source: PLoS Biol. 2006 Dec 26;5(1):e9. doi: 10.1371/journal.pbio.0050009 (PMC1717016; doi:10.1371/journal.pbio.0050009)
Supplement: Supplementary file 5 [file pbio.0050009.sg005.pdf]

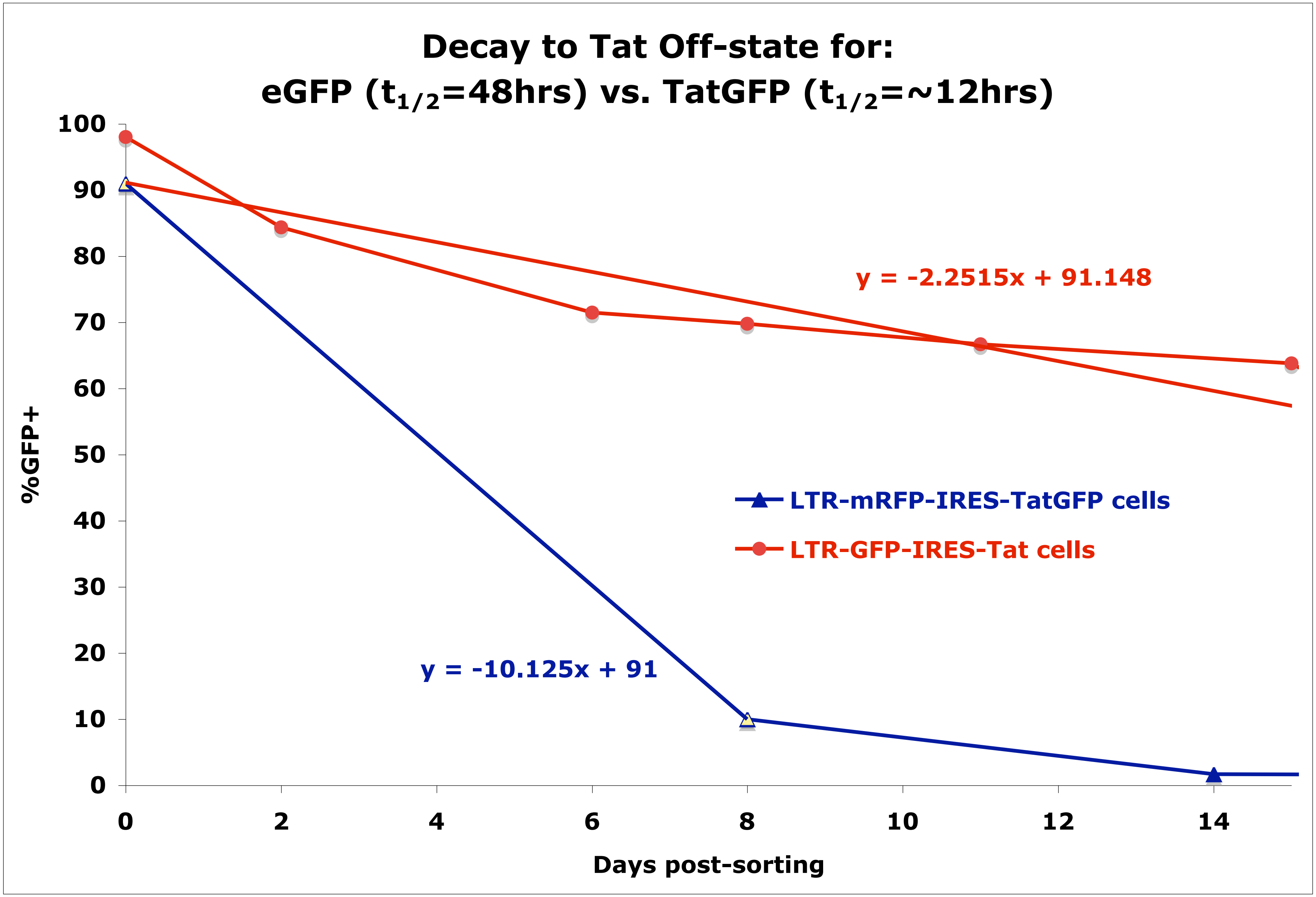
Decay to Tat Off-state for:
eGFP ($t_{1/2}$=48hrs) vs. TatGFP ($t_{1/2}$=~12hrs)
%GFP+
100
90
80
70
60
50
40
30
20
10
0
0
2
4
6
8
10
12
14
Days post-sorting
y = -2.2515x + 91.148
y = -10.125x + 91
LTR-mRFP-IRES-TatGFP cells
LTR-GFP-IRES-Tat cells
